# Perinatal Combinational Exposure to Bisphenol A and a High-Fat Diet Contributes to Transgenerational Dysregulation of Cardiovascular and Metabolic Systems in Mice

**DOI:** 10.3389/fcell.2022.834346

**Published:** 2022-02-24

**Authors:** Juncheng Liu, Maolin Liao, Rongfeng Huang, Yuehua You, Xiaojing Lin, Hong Yang, Lei Fan, Ying Zhong, Xinyu Li, Jibin Li, Xiaoqiu Xiao

**Affiliations:** ^1^ Department of Endocrinology, The First Affiliated Hospital of Chongqing Medical University, Chongqing, China; ^2^ The Chongqing Key Laboratory of Translational Medicine in Major Metabolic Diseases, The First Affiliated Hospital of Chongqing Medical University, Chongqing, China; ^3^ Department of Nutrition and Food Hygiene, School of Public Health and Management, Chongqing Medical University, Chongqing, China; ^4^ Department of Pharmacy, The First Affiliated Hospital of Chongqing Medical University, Chongqing, China

**Keywords:** bisphenol A, cardiovascular system, obesity, offspring, transgenerational inheritance

## Abstract

Both bisphenol A (BPA) and high-fat diet (HFD) exert unfavorable effects on animals and humans; moreover, they could affect the health of their offspring. BPA and HFD often coexist in modern lifestyles; however, the long-term effects of simultaneous exposure of mothers to BPA and HFD during the perinatal period on the cardiovascular and metabolic systems of the offspring remain unclear. This study aimed to examine the effect of simultaneous exposure of mothers to BPA and HFD on the risk of metabolic and cardiovascular abnormalities in offspring. Institute of Cancer Research female mice (F0) were exposed to BPA and fed with HFD before and during gestation until the end of lactation. F0 mice were mated with untreated males to produce the first generation (F1); subsequently, adult F1 males/females were mated with normal females/males to produce the second generation (F2). Combined maternal exposure to BPA and HFD caused myocardial hypertrophy and aortic tunica media thickening as well as increased the cross-sectional area of cardiomyocytes and blood pressure in the matrilineal F2 generation. These cardiovascular changes might be associated with reduced endothelial nitric oxide synthase (eNOS) levels. The patrilineal female F2 was more likely to be obese than the patrilineal male F2. Re-feeding with a HFD showed a more significant weight gain and reduced energy expenditure. However, the aforementioned effects were not observed with exposure to HFD or BPA alone during the perinatal period. Our findings suggest that perinatal combinational exposure to BPA and HFD could cause metabolic and cardiovascular disorders in the offspring, Further, our findings demonstrate that the synergistic effects of HFD and BPA could be transmitted to future generations in a sex-dependent manner.

## Introduction

There has been a worldwide increase in the prevalence of cardiovascular and metabolic diseases, which have made them a grave public health concern (NCD Risk Factor Collaboration (NCD-RisC), 2017; Devos and Menard, 2020). In addition to genetic susceptibility, other risk factors for hypertension and metabolic diseases include lifestyle and environmental factors, especially nutrition (Riccardi et al., 2004; Bergman et al., 2012; Kataria et al., 2017; Dai et al., 2020; Hochsmann et al., 2021). Furthermore, exposure of rodents and humans (F0) to some adverse environmental factors can cause phenotype changes and increased susceptibility to diseases (Kolb and Martin, 2017; Di Ciaula and Portincasa, 2019), with these adverse outcomes being possibly passed down to future generations (F1, F2, etc.) even without direct exposure (Barua and Junaid, 2015). This is considered the transgenerational inheritance of the adverse effects of environmental factors (Krishnan et al., 2018; Schellong et al., 2020). Bisphenol A (BPA) is a typical endocrine-disrupting chemical (EDC) with estrogenic activity. It is the precursor of polycarbonate and epoxy resin and is widely used in food packaging, medical devices, and other daily products (Krishnan et al., 2018; Geens et al., 2021). BPA exerts intergenerational effects on neurodevelopmental abnormalities, hypertension, and metabolic disorders (Wolstenholme et al., 2013; Gawlinski et al., 2021). Exposure to BPA or a high-fat diet (HFD) during pregnancy has similar effects, which increases the risk of hypertension and obesity in offspring (Provvisiero et al., 2016; Bae et al., 2017; Lomas-Soria et al., 2018; Figueiredo et al., 2020). Several large epidemiological studies have demonstrated an association of high exposure levels to BPA or HFD with an increased risk of metabolic and cardiovascular diseases (Han and Hong, 2016; Bao et al., 2020; Choi et al., 2020).

People are often exposed to various adverse factors during their lifetime (Jeon et al., 2015); further, it is more realistic to examine the effects of exposure to multiple, rather than single, factors on health and the offspring (Di Ciaula and Portincasa, 2019). Since high energy diets are very common in modern society, there is a need to determine the effect of combined exposure to a high energy diet and EDCs on health. However, it remains unclear whether the symptoms caused by maternal perinatal period exposure to BPA or HFD in F0, including abnormal blood pressure regulation and metabolic abnormalities, could be passed down to F1, F2, or further generations even without direct exposure to BPA or HFD. Moreover, the effects of perinatal combined exposure to BPA and HFD on the offspring’s health remain unclear.

## Materials and Methods

### Animals

**FIGURE 1:**
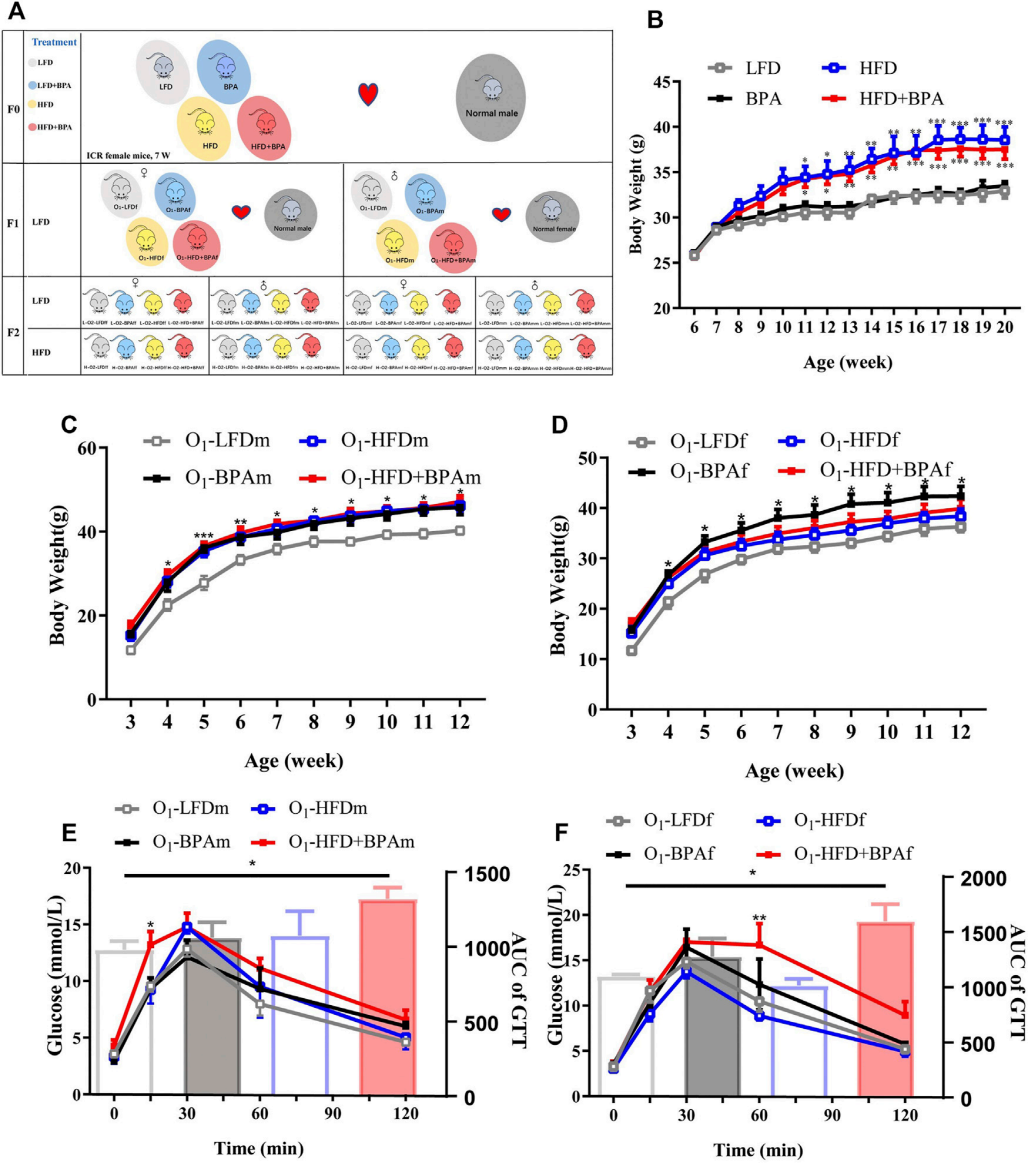
HFD, BPA and their combination affect weight gain, metabolic profile of F0 and F1. **(A)** Schematic diagram of experimental design and animal groups. **(B)** The body weight difference of F0 female exposed to LFD, BPA, HFD, and HFD plus BPA. **(C,E)** Body weight and GTT of F1 male offspring. **(D,F)** Body weight and GTT of F1 female offspring. Data were presented as mean ± SEM and were analyzed by two-way ANOVA with a post hoc test. *p < 0.05, **p < 0.01, ***p < 0.001, BPA, HFD, BPA + HFD vs. LFD.

We purchased 6-week-old Institute of Cancer Research female mice from the Experimental Animal Center of Chongqing Medical University. They adapted to the facility for 1 week before being transferred to the Specific Pathogen Free animal room. All experimental procedures were approved by the Institutional Animal Care and Use Committee of Chongqing Medical University. Based on our previous study, we selected a dose of 500 μg/kg/day (Lv et al., 2017). Mice randomly received four treatments: a control low-fat diet (LFD, with 10% of calories from fat, D12450B, Research Diet, New Brunswick, NJ, United States); BPA (500 μg/kg/day, > 99% purity, Sigma-Aldrich, St Louis, MO, United States); HFD (60% of calories from fat, D12492, Research Diet, New Brunswick, NJ, United States); HFD + BPA (500 μg/kg/day BPA combined with HFD). BPA was dissolved in ethanol, and serial dilutions by phosphate-buffered saline were then performed which allowed for BPA working solution in a final ethanol concentration of 0.01% (BPA final concentration: 50 μg/ml). The solution was freshly prepared for daily gavage. After treatment for 10 weeks, the female mice were mated with age-matched healthy males to produce F1 offspring. The same treatment was maintained during 3-week gestation and lactation periods. All F1 mice received an LFD. The F1 mice were mated with untreated 12-week-old males/females to produce F2 offspring. A vaginal plug indicated successful conception; subsequently, the pregnant mice were housed individually throughout the gestation period until postnatal day 21. The F2 mice received a HFD again at the age of 13 weeks to determine among-group differences in metabolic and cardiovascular phenotypes. Figure 1A shows the experimental design. F2 mice received an LFD for 12 weeks. Subsequently, some mice received a HFD from the 13th week to the 21st week, while the remaining mice received an LFD until the 21st week before being sacrificed. Blood and tissues were collected and stored at −80°C.

### Determination of Serum Hormone Levels

Blood was collected from the retro-orbital sinus puncture before sacrifice. We obtained serum through centrifugation (3,000 g, 15 min, 4°C) for insulin measurement (CSB-E05071m, CUSABIO, Wuhan, China). Analyses were performed in duplicate following the manufacturer’s instructions. The detection range was 15.6–1,000 nIU/ml. In case the measurement was lower than the minimum detection limit, we used the minimum detection limit.

### Measurement of Blood Pressure

We measured the blood pressure rate using a noninvasive computerized tail-cuff blood pressure system (Visitech Systems, Apex, NC, United States). Briefly, we allowed the adapted mice to calm down in a tunnel maintained at 37°C; subsequently, blood pressure was measured in triplicate. The final blood pressure and heart rate were determined by the mean values.

### Echocardiography

Cardiac function was assessed using echocardiography (IE33; Philips, Amsterdam, Netherlands). Mice were anesthetized using 2% isoflurane in 100% oxygen; moreover, images were acquired in the short-axis view to evaluate cardiac function.

### Glucose Tolerance Test

Following fasting (12 h deprivation of diet: 8:00 p.m. to 8:00 a.m.), these mice were intraperitoneally injected with a solution of D-glucose (2 g/kg body weight). We obtained blood samples from the tail vein of the mice at 0 min (just before glucose load), as well as 15, 30, 60, 90, and 120 min after glucose administration. Blood glucose levels were measured using the glucometer, followed by calculation of the area under the curve (AUC).

### Histological Examination

F1 and F2 animals were euthanized at the age of 24 weeks, followed by collection of the aorta and heart after heart perfusion. The aorta and heart were embedded in paraffin and serially sectioned at 4-μm thickness for hematoxylin-eosin staining, followed by observation using the SLIDEVIEW VS200 (Olympus, Tokyo, Japan) under 20× conditions. For morphometric analysis of the aorta, the intima-media thickness was detected using OlyVIA V3.3 software (Olympus, Tokyo, Japan).

### Wheat Germ Agglutinin Staining

The heart was harvested and fixed in 4% paraformaldehyde overnight at 4°C. Tissue sections (5-μm thickness) were stained with wheat germ agglutinin (WGA, L4895, Sigma-Aldrich, St Louis, MO, United States) to assess the cardiomyocyte cross-sectional area in myocardial sections.

### Energy Expenditure

We determined O_2_ consumption, CO_2_ production, and energy expenditure using the PhenoMaster/LabMaster Caging System (TSE System, Bad Homburg, Germany). We individually monitored 22-week-old mice for 48 h; additionally, data were collected at 27-min intervals after being allowed to adapt for 1 day.

### Western Blotting

Aortic rings were used for Western blot analysis. Briefly, we isolated intact aortic rings from the different treated offspring. Subsequently, the rings were homogenized in RIPA buffer containing protease and phosphatase inhibitors. The homogenates were ultrasonicated for 15 s, followed by centrifugation at 4°C for 10 min at 10,000 g. The supernatants were collected to determine the protein levels. Samples with equal protein were loaded and separated on 10% SDS-PAGE. The membranes were incubated with primary antibodies, followed by a secondary horseradish peroxidase-conjugated goat antirabbit antibody. We used the rabbit antibodies against eNOS (32027s, 1:1000, Cell Signaling Technology, Beverly, MA, United States) and anti- β -actin (BM5422, 1:10000, Boster, Wuhan, China). Signals were detected using ECL Western Blotting Detection reagent (Advansta, Menlo Park, CA, United States) and FUSION FX (Vilber Lourmat, Marne La Vallee, France). The band intensity was quantified using FusionCapt Advance FX5 software (Vilber Lourmat, Marne La Vallee, France).

### Statistical Analysis

Results were expressed as the mean ± standard error of the mean. Statistical analyses were performed using GraphPad Prism software version 8 (GraphPad Prism Software Inc., La Jolla, CA, United States). Among-group differences were analyzed using the two-way analysis of variance (ANOVA). Statistical significance was set at *p* < 0.05.

## Results

### HFD, BPA, and Their Combination Affect Weight Gain and Metabolic Profile of F0 and F1 Mice

Figure 1A shows the experimental design and animal groups. There was a significant increase in the bodyweight of female mice in the HFD and HFD + BPA group than of those in the LFD group; moreover, BPA had no direct effect on body weight in F0 mice (Figure 1B). In the F1 generations, males in the HFD, BPA, and HFD + BPA groups were heavier than those in the LFD group (Figure 1C). However, for F1 female mice, only the BPA group weighed heavier than the LFD group (Figure 1D). In the GTT, F1 male mice in the HFD + BPA group showed a significant increase in their glucose levels at 30 min after glucose loading when compared with those in the LFD group (Figure 1E). Contrastingly, F1 female mice in the HFD + BPA group showed a significant increase in glucose levels at 60 min after glucose loading compared with those in the LFD group (Figure 1F).

### Effects of Combined Exposure to BPA and HFD on Body Weight, Glucose Tolerance, Blood Pressure, and Cardiac Structure of Matrilineal Male F2 Mice

**FIGURE 2:**
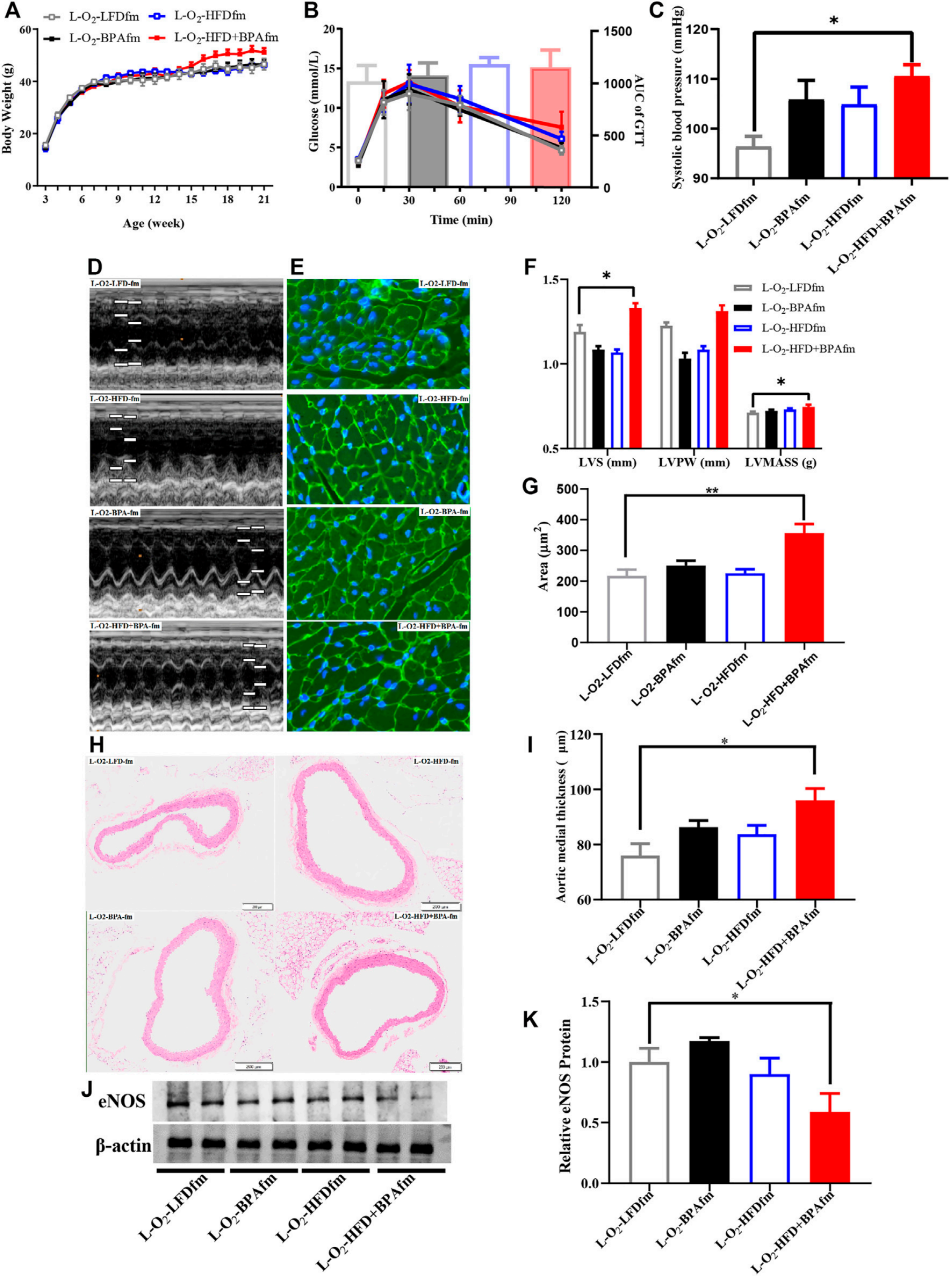
HFD, BPA and their combination affect weight gain, metabolic profile of matrilineal male F2. **(A,B)** The weight and glucose tolerance difference among the four groups of matrilineal male F2 from weaning to 21 weeks. **(C)** The SBP among the four groups of matrilineal male F2. **(D,F)** Echocardiograms among the four groups of matrilineal male F2. **(E,G)** Representative photomicrographs depicting WGA staining of cardiomyocytes and average cardiomyocyte area of matrilineal male F2. **(H,I)** Representative photomicrographs depicting hematoxylin eosin staining of aorta and arterial media thickness. **(J,K)** Western blotting results and quantitative analysis for aorta of matrilineal male F2. Data are mean ± SEM and were analyzed by two-way ANOVA with a posthoc test. *p < 0.05, **p < 0.01, BPA, HFD, BPA + HFD vs. LFD.

There was no significant change in the body weight and glucose tolerance of matrilineal male F2 mice from the 4th week to the 21st week (Figures 2A,B). However, there was a significantly higher systolic blood pressure (SBP) in the L-O_2_-HFD + BPAfm group than in matrilineal male F2 mice in the LFD group (Figure 2C). Echocardiographical analysis revealed significantly higher interventricular septum thickness and left ventricular mass (Figures 2D,F) in the L-O_2_-HFD + BPAfm group than in matrilineal male F2 mice in the LFD group. WGA staining revealed a significant increase in the cardiomyocyte area in the L-O_2_-HFD + BPAfm group than in matrilineal male F2 mice in the LFD group (Figures 2E,G). Additionally, the arterial media thickness (Figures 2H,I) was significantly higher in the L-O_2_-HFD + BPAfm group than in matrilineal male F2 mice in the LFD group. Western blot analysis revealed significantly lower aortic eNOS expression in the L-O_2_-HFD + BPAfm group than in matrilineal male F2 mice in the LFD group (Figures 2J,K).

### Effects of Combined Exposure to BPA and HFD on Body Weight, Glucose Tolerance, Blood Pressure, and Heart Structure of Matrilineal Female F2 Mice

**FIGURE 3:**
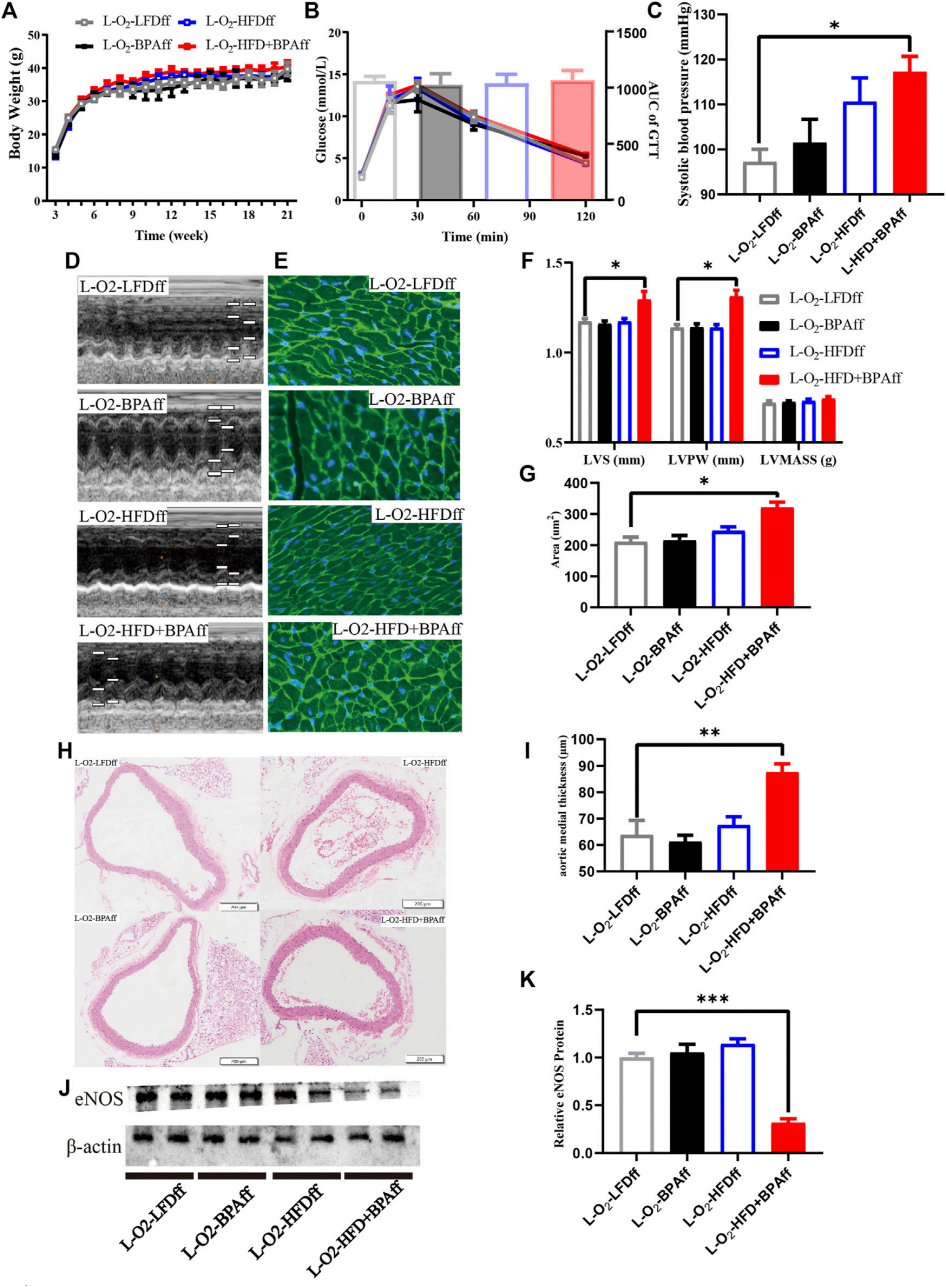
HFD, BPA and their combination affect weight gain, metabolic profile of maternal female F2. **(A,B)** The weight and glucose tolerance difference among the four groups of matrilineal female F2 from weaning to 21 weeks. **(C)** The SBP among the four groups of matrilineal female F2. **(D,F)** Echocardiograms among the four groups of matrilineal female F2. **(E,G)** Representative photomicrographs depicting WGA staining of cardiomyocytes and Average cardiomyocyte area of matrilineal female F2. **(H,I)** Representative photomicrographs depicting hematoxylin eosin staining of aorta and arterial media thickness. **(J,K)** Western blotting results and quantitative analysis for aorta of matrilineal female F2. Data are mean ± SEM and were analyzed by two-way ANOVA with a posthoc test. *p < 0.05. **p < 0.01. ***p < 0.001, BPA, HFD, BPA + HFD vs LFD.

There was no significant difference in body weight and glucose tolerance of matrilineal male F2 from weaning to 21 weeks (Figures 3A,B). SBP was significantly higher in the L-O_2_-HFD + BPAfm group than in matrilineal female F2 mice in the LFD group (Figure 3C). Echocardiography revealed significantly higher interventricular septum thickness and left ventricular posterior wall thickness (Figures 3D,F) were significantly thicker in the L-O_2_-HFD + BPAfm group than in matrilineal female F2 mice in the LFD group. WGA staining revealed a significantly higher cardiomyocyte area in the L-O_2_-HFD + BPAff group than in matrilineal female F2 mice in the LFD group (Figures 3E,G). The L-O_2_-HFD + BPAff group had a significantly higher arterial media thickness (Figures 3H,I) than matrilineal female F2 mice in the LFD group. Western blot analysis revealed significantly lower aortic eNOS expression in the L-O_2_-HFD + BPAff group than in matrilineal female F2 mice in the other three groups (Figures 3J,K).

### HFD, BPA, and Their Combination Affect Metabolic Profile and Blood Pressure of Patrilineal F2 Mice

**FIGURE 4:**
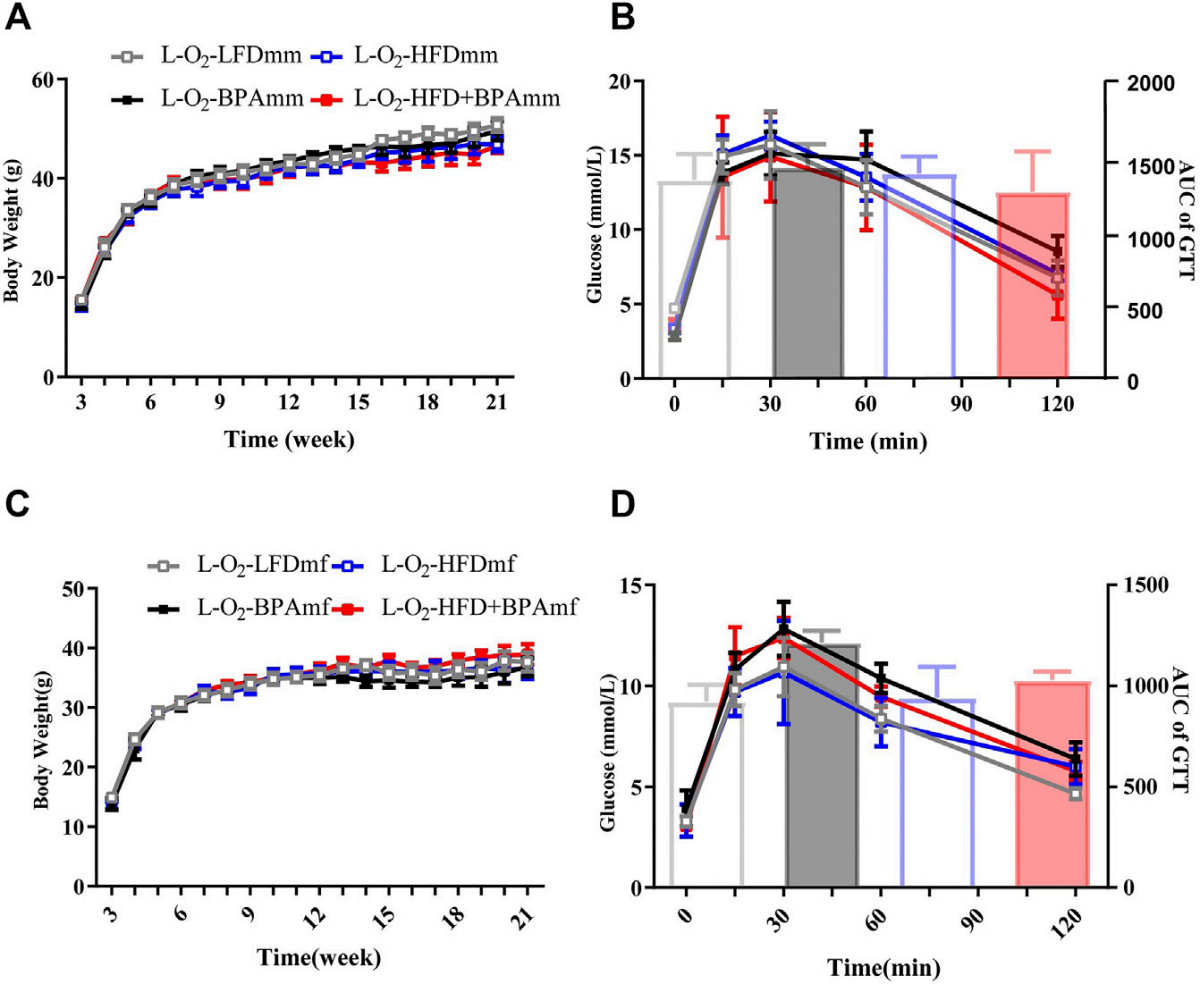
HFD, BPA and their combination affect weight gain, glucose of patrilineal F2 with low fat diet. **(A,B)** Body weight and glucose tolerance of patrilineal male F2. **(C,D)** Body weight and glucose tolerance of patrilineal female F2. Data are mean ± SEM and were analyzed by two-way ANOVA with a posthoc test.

**FIGURE 5:**
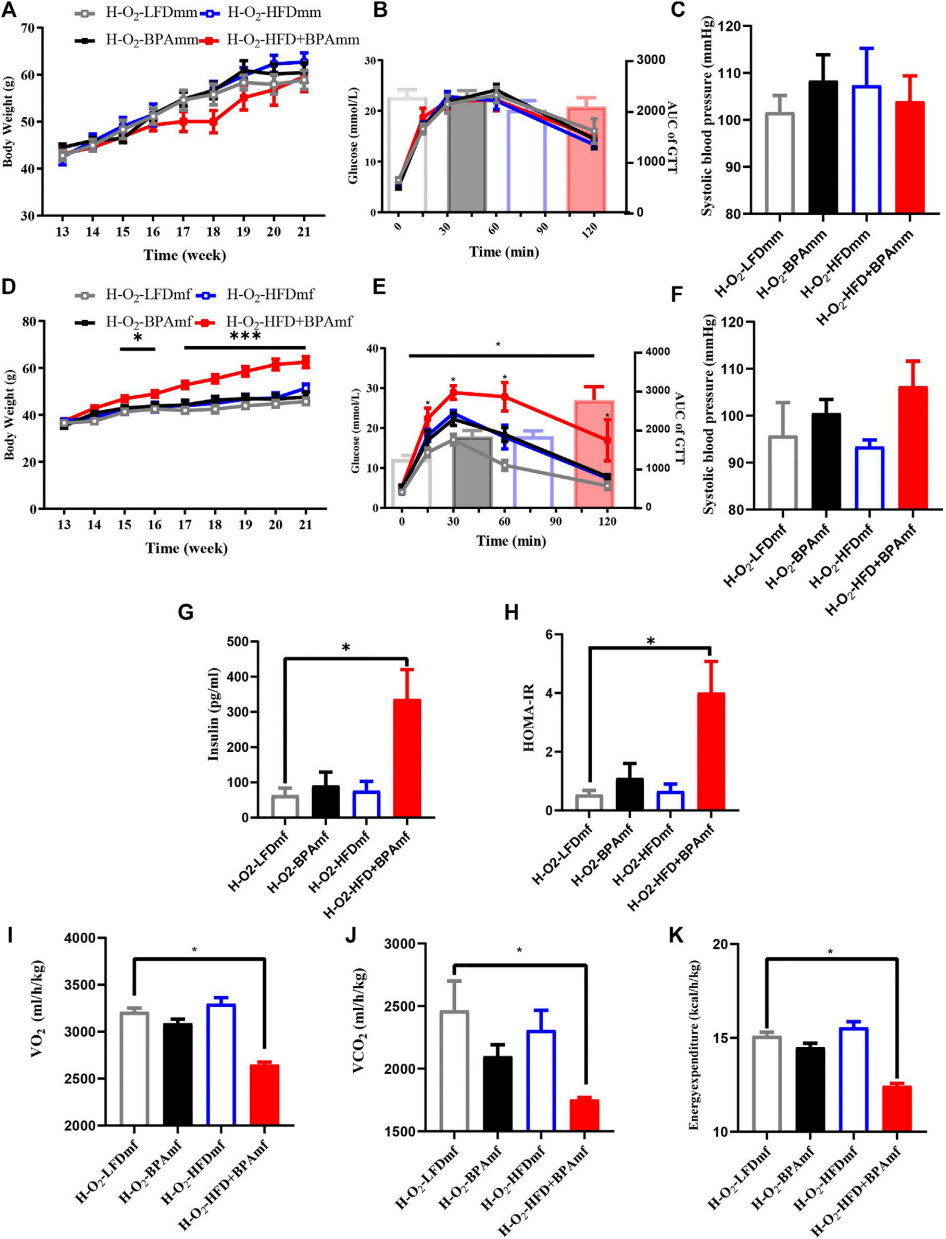
HFD, BPA and their combination affect weight gain, metabolic profile and blood pressure of patrilineal F2 with HFD. **(A–C)** Body weight, glucose tolerance and systolic blood pressure of patrilineal male F2 with HFD. **(D–F)** Body weight, glucose tolerance and systolic blood pressure of females and males of patrilineal female F2 with HFD. **(G,H)** Serum insulin concentration and HOMA-IR of patrilineal female F2 with HFD. **(I–K)** metabolism cage of patrilineal female F2 with HFD. Data are means ± SEMs and were analyzed by two-way ANOVA with a posthoc test. *p < 0.05, ***p < 0.001, BPA, HFD, BPA + HFD vs. LFD.

There were no significant sex differences in body weight and glucose tolerance in adult patrilineal F2 mice (Figures 4A–D). Adult patrilineal F2 mice were fed a HFD from the 12th to 21st week to examine changes in metabolic or cardiovascular phenotypes after re-administration of a HFD. However, there were no significant changes in body weight and glucose tolerance in patrilineal male F2 mice (Figures 5A–C). However, there was a significant increase in the body weight and impaired glucose tolerance in HFD-fed adult patrilineal female F2 mice from ancestors exposed to both HFD and BPA compared with their counterparts from ancestors exposed to an LFD diet during the perinatal period (H-O2-HFD + BPAmf vs. H-O2-LFDmf, Figures 5D,E). There was no significant change in blood pressure among HFD-fed patrilineal female F2 mice (Figures 5C,F). Female patrilineal F2 mice showed significantly higher serum insulin levels and homeostatic model assessment–insulin resistance (HOMA-IR) in the H-O2-HFD + BPAmf group than in the LFD group (Figures 5G,H). Metabolic cage testing revealed significantly decreased energy expenditure, oxygen intake, and carbon dioxide production in the H-O2-HFD + BPAfm group than in the H-O2-LFDmf group (Figures 5I–K). These findings suggested that decreased energy expenditure may contribute to increased body weight, impaired glucose tolerance, and insulin resistance in the H-O2-HFD + BPAmf group.

### Effects of Combined Exposure to BPA and HFD on Insulin of Matrilineal F2 and Their Metabolic Profile for Exposing HFD Again

**FIGURE 6:**
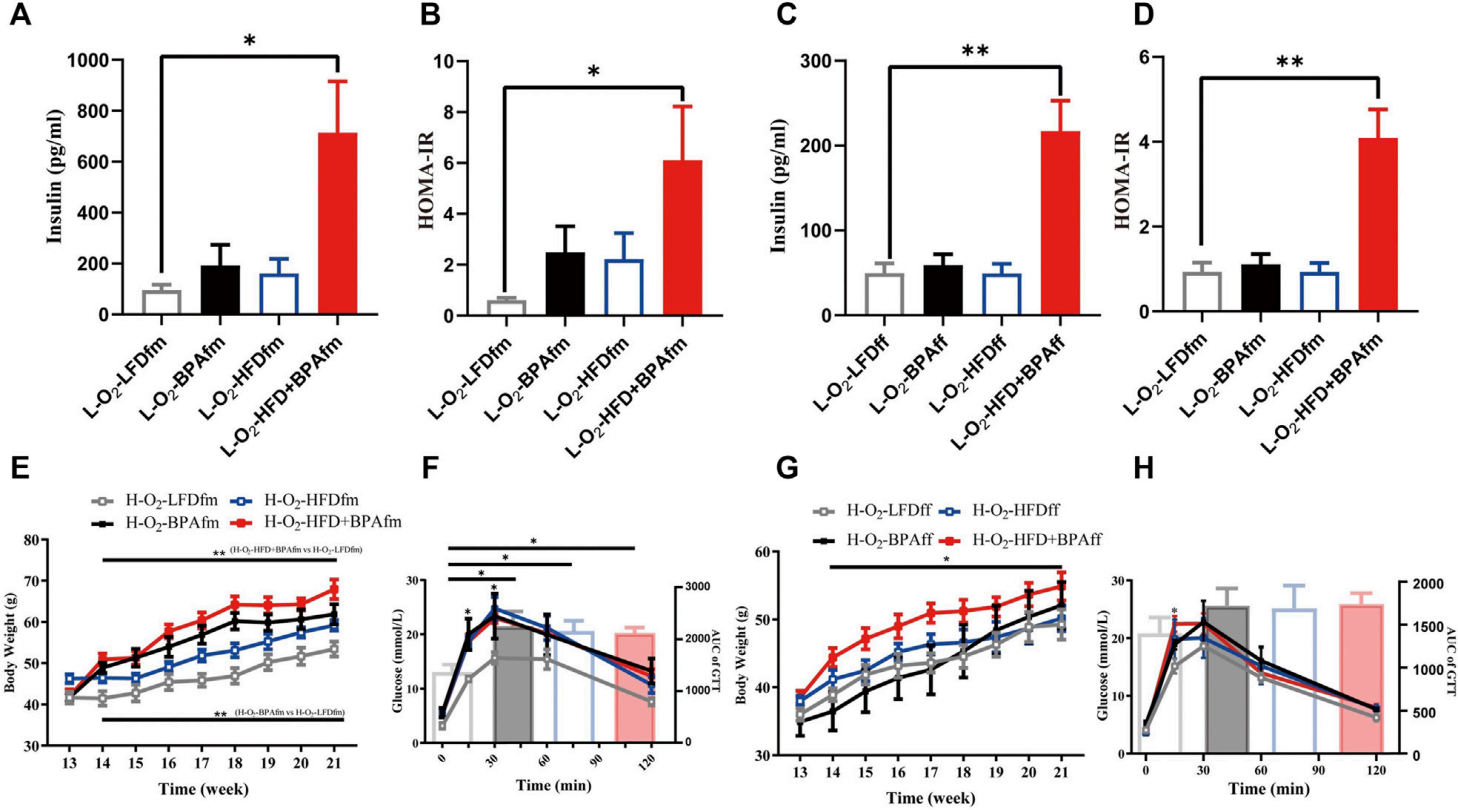
Effects of combined exposure to BPA and HFD on insulin of matrilineal F2 and their metabolic profile for exposing HFD again. **(A–D)** Serum insulin concentration and insulin resistance index. **(E–H)** Body weight and glucose tolerance of maternal F2 when exposed to HFD again. Data are means ± SEMs and were analyzed by two-way ANOVA with a posthoc test. *p < 0.05, **p < 0.01, BPA, HFD, BPA + HFD vs LFD.

The L-O2-HFD + BPAff group showed significantly higher serum insulin levels and HOMA-IR in the LFD group (Figures 6A–D). Adult matrilineal F2 mice were fed a HFD from the 12th to 21st week to examine the metabolic profile, which revealed significantly increased body weight and impaired glucose tolerance. However, these effects were mainly attributed to BPA or HFD rather than their synergistic effect (Figures 6E,F). There was a significant increase in the bodyweight of HFD-fed adult matrilineal female F2 mice from ancestors exposed to both HFD and BPA compared with counterparts from ancestors exposed to an LFD diet during the perinatal period (Figure 6G). However, the AUC did not reveal a significant change in glucose tolerance; however, the H-O2-HFD + BPAff group showed a significant increase in glucose levels at 15 min after glucose loading (Figure 6H).

## Discussion

This study examined the transgenerational inheritance of adverse outcomes after combinational exposure of female mice (F0) to BPA and HFD during the perinatal period, with a particular focus on metabolic and cardiovascular changes in F2. In contrast to studies on the direct toxicity of exposure to BPA or HFD (Lecoutre et al., 2016; Bansal et al., 2017; Tain et al., 2017; Lomas-Soria et al., 2018; Choi et al., 2020; Filardi et al., 2020), we examined the long-term and synergistic effects of BPA and HFD. Notably, we observed that patrilineal female F2 mice mainly showed metabolic dysfunction signs, including increased weight gain, impaired glucose tolerance, and insulin resistance. Contrastingly, matrilineal F2 mice primarily showed cardiovascular changes, including increased blood pressure, cardiac remodeling, and aorta intimal thickening. This suggests that the adverse effects of combined exposure to BPA and HFD during the perinatal period could be sex-specific. Other studies have reported these sex differences, which indicates that the outcomes of perinatal exposure to adverse factors could have sex-dependent effects on offspring (Tain et al., 2017; Lomas-Soria et al., 2018; Sarker et al., 2018). Additionally, compared with exposure to the LFD, HFD, or BPA, exposure to both BPA and HFD decreased aortic eNOS expression in F2 mice, which indicates a significant synergistic effect of combined exposure to HFD and BPA. Further, this demonstrates that exposure to multiple adverse factors during the perinatal period has a more profound impact than exposure to a single adverse factor (Hsu et al., 2019). Moreover, patrilineal female mice, as well as matrilineal female and male mice, showed increased serum insulin levels and insulin resistance, which is consistent with previous reports that BPA or HFD exposure can cause insulin resistance (Naville et al., 2019; Farrugia et al., 2021; Gao et al., 2021; Huang et al., 2021; Yang et al., 2021). This may in turn contribute to metabolic- and cardiovascular-related phenotypic changes. Finally, we found that exposure to HFD or BPA alone did not significantly affect F2 mice; however, combined exposure to BPA and HFD yielded a synergistic effect, which caused significant and long-term changes in metabolic and cardiovascular phenotypes. Our findings demonstrated that early-life exposure to multiple factors may synergistically lead to more severe adverse outcomes, with these changes being passed on to the future generation. In summary, the toxicity of BPA could be significantly aggravated by the co-presence of HFD, which suggests environmental pollutants with the high energy diets may exert more severe and longer adverse impacts on human health and therefore should be further addressed in future work.

The mechanisms underlying the induction of metabolic and cardiovascular changes after early-life exposure to BPA and HFD remain unclear. Maternal exposure to adverse factors during the perinatal period could alter the intrauterine environment, which increases the risk of chronic disease in offspring (Nivoit et al., 2009; Vickers, 2014; Argyraki et al., 2019). For example, *in utero* malnutrition can cause changes in the fetal conditions, which promote dysfunction of certain organs in later life. These effects could be multigenerational in some cases (Bale, 2014; Picton and Balen, 2019). Transgenerational transmission of metabolic dysfunction is potentially related to epigenetics. For example, maternal exposure to BPA causes pancreatic islet dysfunction in the next generation, which may be associated with methylation of pancreatic islet genes Igf2 and Esr1 (Bansal et al., 2017). Maternal exposure to a HFD causes metabolic disorders in the offspring through epigenetics (Fleming et al., 2018). Future studies should clarify the involvement of epigenetics in our model. Moreover, our findings demonstrated sexual dimorphism. Human and mouse studies have showed sexual dimorphism in susceptibility to metabolic disease (Mauvais-Jarvis, 2015; Kautzky-Willer et al., 2016; Monrroy et al., 2019; Lefebvre and Staels, 2021). These sex differences could be attributed to differences in energy metabolism and the actions of sex hormones (Xiao et al., 2008; Tramunt et al., 2020). Furthermore, prenatal effects may be affected by sex-specific differences. There are sex differences in epigenetic regulation of the fetus and placenta, which could contribute to the differential sensitiveness of males and females in metabolic and cardiovascular disease (Kautzky-Willer et al., 2016).

In our study, when only the F0 generation was directly exposed to unfavorable factors, compared with the LFD group, maternal F2 mice in the HFD + BPA group showed significant cardiovascular changes. Contrastingly, for patrilineal F2 female mice who were re-exposed to a HFD, the HFD + BPA group showed more obvious metabolic disorders. However, for matrilineal F2 mice, re-exposure to a HFD did not show a synergistic effect of exposure to HFD and BPA on the metabolic phenotype. Therefore, the weight gain, insulin resistance, hypertension, and cardiac hypertrophy in F2 may result from the direct transgenerational inheriting effects caused by the synergistic effects of BPA and HFD exposure in F0 generation. These results demonstrate that perinatal exposure of the F0 generation to BPA and HFD can cause harmful changes in metabolic and cardiovascular systems in their F2 offspring.

This study has several limitations. First, we mainly focused on the phenotype, and the mechanism should be further explored. Thus, our future studies will examine changes in mitochondrial function, reactive oxygen species (ROS) levels and global gene expression profile and clarify whether epigenetic mechanism, especially DNA methylation will influence the effect of maternal adverse factors exposure on metabolism and cardiovascular system of the offspring. Second, we did not explore the effects of different doses of BPA exposure on offspring to determine whether they would lead to different phenotypes in adult offspring.

Our findings demonstrated that combinational exposure of female mice to HFD and BPA during the perinatal period can cause susceptibility to insulin resistance, obesity, impaired glucose tolerance, increased blood pressure, cardiac hypertrophy, and impaired endothelial function in their F2 offspring. In addition, these inherited transgenerational abnormalities showed a sex-specific pattern. Our findings demonstrate the requirement of adjusting lifestyle and alleviating exposure to environmental EDSs during pregnancy to reduce the risk of metabolic and cardiovascular diseases in the offspring.

## Data Availability

The original contributions presented in the study are included in the article/supplementary material, further inquiries can be directed to the corresponding author.
